# Association of Time to Surgery After COVID-19 Infection With Risk of Postoperative Cardiovascular Morbidity

**DOI:** 10.1001/jamanetworkopen.2022.46922

**Published:** 2022-12-14

**Authors:** John M. Bryant, Christina S. Boncyk, Kimberly F. Rengel, Vivian Doan, Connor Snarskis, Matthew D. McEvoy, Karen Y. McCarthy, Gen Li, Warren S. Sandberg, Robert E. Freundlich

**Affiliations:** 1 Department of Anesthesiology, Vanderbilt University Medical Center, Nashville, Tennessee; 2 Department of Surgery, Vanderbilt University Medical Center, Nashville, Tennessee; 3 Department of Biomedical Informatics, Vanderbilt University Medical Center, Nashville, Tennessee

## Abstract

**Importance:**

The time interval between COVID-19 infection and surgery is a potentially modifiable but understudied risk factor for postoperative complications.

**Objective:**

To examine the association between time to surgery after COVID-19 diagnosis and the risk of a composite of major postoperative cardiovascular morbidity events within 30 days of surgery.

**Design, Setting, and Participants:**

This single-center, retrospective cohort study was conducted among 3997 adult patients (aged ≥18 years) with a previous diagnosis of COVID-19, as documented by a positive polymerase chain reaction test result, who were undergoing surgery from January 1, 2020, to December 6, 2021. Data were obtained through Structured Query Language access of an existing perioperative data warehouse. Statistical analysis was performed March 29, 2022.

**Exposure:**

The time interval between COVID-19 diagnosis and surgery.

**Main Outcomes and Measures:**

The primary outcome was the composite occurrence of major cardiovascular comorbidity, defined as deep vein thrombosis, pulmonary embolism, cerebrovascular accident, myocardial injury, acute kidney injury, and death within 30 days after surgery, using multivariable logistic regression.

**Results:**

A total of 3997 patients (2223 [55.6%]; median age, 51.3 years [IQR, 35.1-64.4 years]; 667 [16.7%] African American or Black; 2990 [74.8%] White; and 340 [8.5%] other race) were included in the study. The median time from COVID-19 diagnosis to surgery was 98 days (IQR, 30-225 days). Major postoperative adverse cardiovascular events were identified in 485 patients (12.1%). Increased time from COVID-19 diagnosis to surgery was associated with a decreased rate of the composite outcome (adjusted odds ratio, 0.99 [per 10 days]; 95% CI, 0.98-1.00; *P* = .006). This trend persisted for the 1552 patients who had received at least 1 dose of COVID-19 vaccine (adjusted odds ratio, 0.98 [per 10 days]; 95% CI, 0.97-1.00; *P* = .04).

**Conclusions and Relevance:**

This study suggests that increased time from COVID-19 diagnosis to surgery was associated with a decreased odds of experiencing major postoperative cardiovascular morbidity. This information should be used to better inform risk-benefit discussions concerning optimal surgical timing and perioperative outcomes for patients with a history of COVID-19 infection.

## Key Points

**Question:**

What is the association of time to surgery after COVID-19 diagnosis with the risk of postoperative cardiovascular complications?

**Findings:**

In this cohort study of 3997 adult patients, increasing time from COVID-19 diagnosis to surgery was associated in a time-dependent fashion with a decreasing rate of a composite outcome of major cardiovascular events.

**Meaning:**

This study suggests that delaying surgery after COVID-19 infection was associated with decreasing postoperative cardiovascular morbidity and should be a factor in shared decision-making between clinicians and patients.

## Teaser

This cohort study examines the association between time to surgery after COVID-19 diagnosis and the risk of a composite of major postoperative cardiovascular morbidity events within 30 days of surgery.

## Introduction

More than 28 million surgical procedures have been cancelled or postponed because of active COVID-19 infections.^1^ Recent evidence has shown increased mortality for surgical patients infected with COVID-19 within 6 weeks of their operation.^2^ Based on available data, the American Society of Anesthesiologists (ASA) and Anesthesia Patient Safety Foundation (APSF) jointly recommend delaying surgery for 4 to 12 weeks after diagnosis, with the variable timeline being based on the severity of COVID-19 and vaccination status.^3^

Although COVID-19 primarily affects the pulmonary system, it is also known to cause endothelial dysfunction, vascular inflammation, and sweeping changes in the coagulation cascade, all of which are thought to result in higher rates of deep vein thrombosis (DVT), pulmonary embolus (PE), and cerebrovascular accident (CVA).^4^ Furthermore, regional inflammation and cytokine release have been linked with pulmonary complications, myocardial injury, and acute kidney injury (AKI).^5^ Taken together, these associations present a substantial potential mortality risk for surgical patients, who experience additional inflammatory activation as a result of their operation.

Although multiple studies have examined the overall risk of mortality after COVID-19 diagnosis and surgery, the association of timing with mortality warrants further investigation. We sought to address the function of time from COVID-19 diagnosis to surgery, hypothesizing that shorter time intervals between COVID-19 infection and surgery would be associated with an increased risk of postoperative cardiovascular morbidity. In addition, we sought to expand on prior reports of perioperative outcomes after COVID-19 infection and to assess the risk of adverse outcomes including DVT, PE, CVA, myocardial injury, and AKI within 30 days of surgery. Finally, we evaluated for variation in these associations based on patient vaccination status.

## Methods

The study protocol was approved by the Vanderbilt University Medical Center (VUMC) institutional review board. A waiver of the requirement for written informed consent was granted because the study was of minimal risk, the waiver was believed to not adversely affect participants, and the research could not practicably be carried out without the waiver. This study adheres to the Strengthening the Reporting of Observational Studies in Epidemiology (STROBE) reporting guideline.^6^

### Study Population

We included all adult patients (aged ≥18 years) with a history of confirmed COVID-19, as documented by a positive polymerase chain reaction (PCR) test result, who were undergoing surgery at VUMC between January 1, 2020, and December 6, 2021. Patient data were extracted from the VUMC perioperative data warehouse (Nashville, Tennessee) using Structured Query Language.

### Primary Outcome and Definitions

The primary outcome was the composite occurrence of DVT, PE, CVA, myocardial injury, AKI, or death within 30 days of surgery. We identified DVT or PE as the presence of both (1) *International Statistical Classification of Diseases and Related Health Problems, Tenth Revision* (*ICD-10*) codes (I82 subset for DVT and I26 subset for PE) and (2) DVT or PE radiologic scan in the hospital encounter. Cerebrovascular accident was defined as the presence of both (1) CVA-specific imaging (noncontrast computed tomography, head and neck computed tomography angiography, magnetic resonance imaging, or magnetic resonance angiography) and (2) a postoperative neurology consultation placement. We considered including *ICD-10* codes for stroke and other imaging modalities in the definition; however, both were excluded after extensive manual review and adjudication owing to a high amount of interobserver variability between medical record documenters, as well as the high rate of false positives caused by the existence of a preoperative CVA diagnosis. Myocardial injury was defined as an increase in the level of serum troponin of 0.04 ng/mL or more (to convert to migrograms per liter, multiply by 1.0).^7^ Acute kidney injury was defined using the Kidney Disease: Improving Global Outcomes (KDIGO) criteria, extended to 30 days, rather than 7 days after surgery: an increase in serum creatinine by 0.3 mg/dL or more (to convert to micromoles per liter, multiply by 88.4) within 48 hours or an increase in serum creatinine to 1.5 times baseline or more.^8^ For patients in whom a preoperative serum creatinine baseline was unknown, an estimated baseline value was inputted using the simplified Modification of Diet In Renal Disease formula based on age, sex, and ethnicity.^9^ The urine output portion of the KDIGO criteria was not included in the definition of acute kidney injury.^10^

### Statistical Analysis

Statistical analysis was performed March 29, 2022. Demographic, clinical, and procedural variables were used to characterize the study population, with mean (SD) values or median values and IQRs for continuous variables and with counts and proportions for categorical variables, as appropriate.

The primary objective of the analysis was to examine the association between the time interval from COVID-19 diagnosis to surgery and a composite outcome of DVT, PE, CVA, myocardial injury, AKI, or death within 30 days after surgery using multivariable logistic regression. We screened 31 Elixhauser comorbidities,^11^ age, sex, race (categorized into 3 groups: African American or Black, White, and other [defined as all races other than African American or Black and White]), American Society of Anesthesiologists (ASA) physical status classification, ASA emergency status, surgical case acuity (elective vs emergency), duration of surgery, intracranial neurosurgical procedure (given increased risk of CVA in this cohort^12^), and urologic procedure (given increased risk of AKI in this cohort^13^) as covariates for potential confounding associations. Because higher incidences of adverse events have been reported among patients in minority groups after a COVID-19 infection,^14,15^ race was included in this study as a potential covariate. Race was recorded as the patient reported race in the electronic health record at our institution. A least absolute shrinkage and selection operator approach was applied to screen covariates for inclusion in the multivariable regression model. In addition, each postoperative outcome was evaluated individually with separate logistic regression analysis. Associations were summarized using odds ratios (ORs) and 95% CIs and tested using a Wald-type test with 5% type I error rate.

We conducted a prespecified sensitivity analysis with the same parameters but excluded patients who experienced the competing outcome of 30-day mortality. We also performed several post hoc sensitivity analyses. First, in response to the ASA-APSF guidance (issued after our prespecified analysis was finalized), which was based largely on the incidence of postoperative pulmonary complications, we analyzed a secondary composite outcome that added the occurrence of postoperative pulmonary complications to our primary composite outcome. Postoperative pulmonary complications were defined as the occurrence of any of the following events within 30 days after surgery: pneumonia, respiratory failure, aspiration pneumonitis, iatrogenic pulmonary embolism, pulmonary infarction, iatrogenic pneumothorax, pulmonary congestion and hypostasis, and other respiratory complications. All postoperative pulmonary complications were identified by *ICD-10* codes.

An additional analysis was performed to evaluate the association between exposure variable and the risk of primary composite outcome for patients with symptomatic COVID-19 and those with asymptomatic COVID-19, separately. The clinician documented the presence of symptoms at the time of specimen collection using a structured data element.

Finally, an analysis was performed to compare vaccinated patients with unvaccinated patients. A vaccinated patient was identified as a patient who received at least 1 injection of the US Food and Drug Administration–approved or –authorized vaccine, including the BNT162b2 (Pfizer-BioNtech) vaccine, the mRNA-1273 (Moderna) vaccine, and the Ad26.COV2.S (Johnson & Johnson and Janssen) vaccine, prior to the surgery.

A 2-sided hypothesis test with *P* < .05 was used to indicate statistical significance. All statistical programming was implemented in SAS, version 9.4 (SAS Institute Inc).

## Results

We identified 3997 eligible patients (2223 [55.6%]; median age, 51.3 years [IQR, 35.1-64.4 years]; 667 [16.7%] African American or Black; 2990 [74.8%] White; and 340 [8.5%] other race) who underwent surgery between January 1, 2020, and December 6, 2021, with a positive SARS-CoV-2 PCR test result prior to surgery (eAppendix in Supplement 1). The median time from COVID-19 diagnosis to surgery was 98 days (IQR, 30-225 days), and 1394 patients (34.9%) underwent surgery within 7 weeks of a confirmed COVID-19 diagnosis. Baseline characteristics are outlined in Table 1.

**Table 1.** Demographic Characteristics of the Study Sample

Characteristic	Patients (N = 3997)
Age, median (IQR), y	51.3 (35.1-64.4)
BMI, median (IQR)	29.0 (25.0-34.0)
Sex, No. (%)	
Female	2223 (55.6)
Male	1774 (44.4)
Race, No. (%)	
African American or Black	667 (16.7)
White	2990 (74.8)
Other^a^	340 (8.5)
ASA physical status classification, No. (%)	
1	193 (4.8)
2	1325 (33.1)
3	1994 (49.9)
4	469 (11.7)
5	15 (0.4)
6	1 (0.03)
ASA emergency, No. (%)	277 (6.9)
Case acuity, No. (%)	
Elective	3701 (92.6)
Emergency	296 (7.4)
Duration of surgery, median (IQR), h	1.5 (0.7-3.0)
Time from confirmed COVID-19 diagnosis to surgery, median (IQR), d	98 (30-225)
Surgical service, No. (%)	
Cardiac	230 (5.8)
Obstetrics and gynecology	384 (9.6)
Neurosurgery	103 (2.6)
Urology	232 (5.8)
Head and neck	417 (10.4)
Vascular and thoracic	133 (3.3)
Emergency general surgery and trauma	124 (3.1)
Gastroenterology	1003 (25.1)
General abdominal	370 (9.3)
Orthopedic	571 (14.3)
Other	430 (10.8)
30-Day reoperation rate, No. (%)	215 (5.4)
Comorbid events, No. (%)	
Deep vein thrombosis	61 (1.5)
Pulmonary embolus	16 (0.4)
Cerebrovascular accident	29 (0.7)
Myocardial injury	116 (2.9)
Acute kidney injury	363 (9.1)
30-Day mortality, No. (%)	79 (2.0)
Incidence of primary composite outcome, No. (%)	485 (12.1)

Abbreviations: ASA, American Society of Anesthesiologists; BMI, body mass index (calculated as weight in kilograms divided by height in meters squared).

^a^ Defined as all races other than African American or Black and White.

In our cohort, we identified 61 patients (1.5%) who developed DVT, 16 (0.4%) who met criteria for PE, 29 (0.7%) with CVA, 116 (2.9%) with myocardial injury, and 363 (9.1%) with AKI within 30 days of surgery (Table 1). In addition, 79 patients (2.0%) died within 30 days after surgery. The overall incidence of the primary composite outcome was 12.1% (n = 485).

From the results of the multivariable logistic regression model, we found that increasing time from positive SARS-CoV-2 test result to surgery was associated with a decreasing rate of the primary composite outcome (adjusted OR [aOR], 0.99 [per 10 days]; 95% CI, 0.98-1.00; *P* = .006). Older age (aOR, 1.13 [per 10 years of age]; 95% CI, 1.05-1.22; *P* = .002), male sex (aOR, 1.51; 95% CI, 1.18-1.93; *P* < .001), Black or African American race (aOR, 2.01 [vs White race]; 95% CI, 1.50-2.70; *P* < .001), higher ASA classification (aOR, 2.43 [per 1 level]; 95% CI, 1.97-2.99; *P* < .001), ASA emergency status (aOR, 1.49; 95% CI, 1.02-2.17; *P* = .04), urologic procedure (aOR, 1.98; 95% CI, 1.37-2.87; *P* < .001), and 8 Elixhauser comorbidities (cardiac arrhythmias, neurodegenerative disorders, kidney failure, lymphoma, solid tumor, coagulopathy, weight loss, and fluid and electrolyte disorders) were independently associated with an increased likelihood of the primary outcome measure (Table 2). The distribution of estimated risk for the continuous variable of time was analyzed graphically using splines (Figure). Similar associations between the time from positive SARS-CoV-2 test result to surgery and primary composite outcome were detected from analyses of the individual outcomes of myocardial injury (aOR, 0.97 [per 10 days]; 95% CI, 0.95-0.99; *P* = .004) (eTable 1 in Supplement 1), AKI (aOR, 0.99 [per 10 days]; 95% CI, 0.98-1.00; *P* = .04) (eTable 2 in Supplement 1), and 30-day mortality (aOR, 0.96 [per 10 days]; 95% CI, 0.93-0.99; *P* = .007) (eTable 3 in Supplement 1).

**Table 2.** Multivariable Logistic Regression Models for Estimating the Risk of Postoperative Adverse Outcomes^a^

Variables	aOR (95% CI)	*P* value
Time interval from COVID-19–positive diagnosis to surgery (per 10-d increase)	0.99 (0.98-1.00)	.006
Age (per 10 y older)	1.13 (1.05-1.22)	.002
Male sex	1.51 (1.18-1.93)	<.001
Black or African American race (vs White race)	2.01 (1.50-2.70)	<.001
ASA classification (per 1 level higher)	2.43 (1.97-2.99)	<.001
ASA emergency status	1.49 (1.02-2.17)	.04
Urologic procedure	1.98 (1.37-2.87)	<.001
Elixhauser comorbidities		
Cardiac arrhythmias	2.02 (1.54-2.64)	<.001
Neurodegenerative disorders	1.93 (1.14-3.27)	.02
Kidney failure	2.67 (2.02-3.56)	<.001
Lymphoma	3.04 (1.05-8.78)	.04
Solid tumor	2.03 (1.41-2.91)	<.001
Coagulopathy	2.38 (1.59-3.54)	<.001
Weight loss	1.91 (1.29-2.81)	.001
Fluid and electrolyte disorders	2.71 (2.03-3.63)	<.001

Abbreviations: aOR, adjusted odds ratio; ASA, American Society of Anesthesiologists.

^a^ Primary composite outcome of postoperative deep vein thrombosis, pulmonary embolus, cerebrovascular accident, myocardial injury, acute kidney injury, and death within 30 days after surgery.

**Figure.**
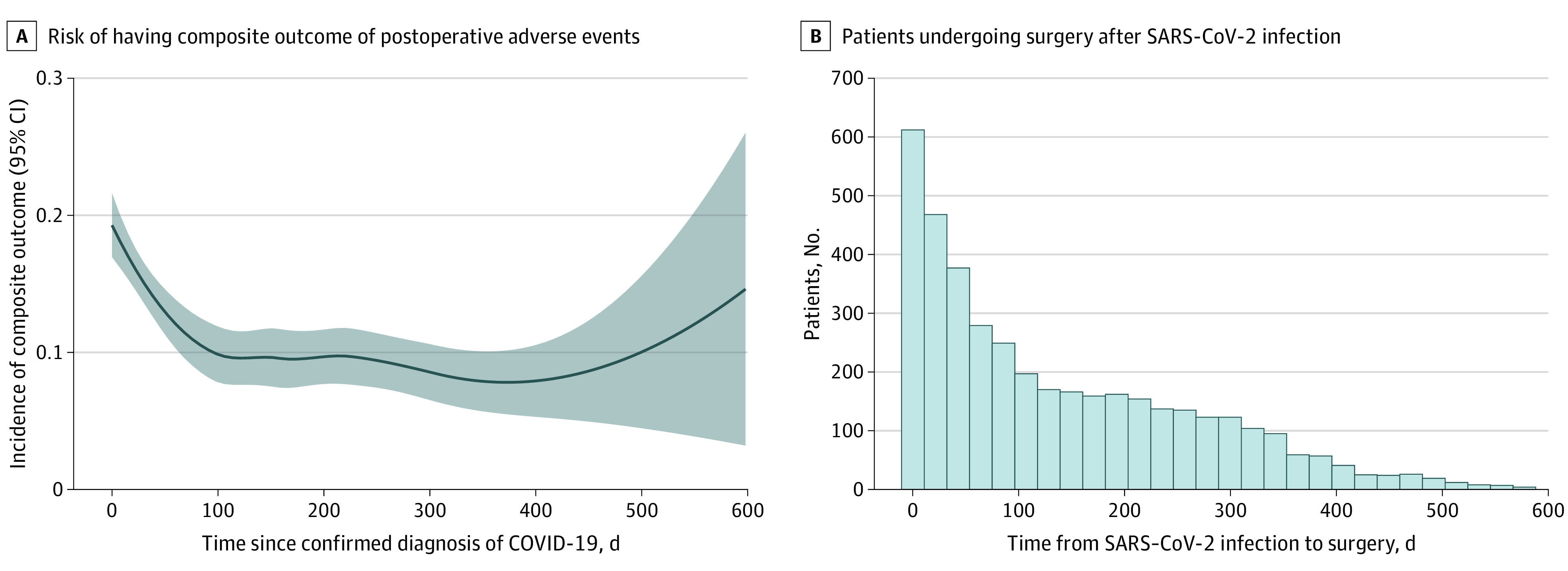
Risk of Postoperative Adverse Outcomes Shown by Level of Continuous Patient Characteristics A, Incidence of composite outcome. Shaded regions indicate 95% CIs. B, Patients undergoing surgery after SARS-CoV-2 infection.

A sensitivity analysis excluding the 79 instances of mortality was congruent with the primary analysis (aOR, 0.99 [per 10-day increase]; 95% CI, 0.98-1.00; *P* = .007) (eTable 4 in Supplement 1). A sensitivity analysis including the 244 patients (6.1%) with postoperative pulmonary complications resulted in a secondary composite outcome incidence rate of 14.7% (n = 588), with findings consistent with the primary analysis (aOR, 0.99 [per 10 days]; 95% CI, 0.98-1.00; *P* = .002) (eTable 5 in Supplement 1).

At the time of testing, 2350 patients (58.8%) had symptomatic infections, and 1539 (38.5%) had asymptomatic infections; the remaining 108 cases (2.7%) did not have structured data on whether they were symptomatic. Consistent with the primary analysis, increasing duration of time from a positive SARS-CoV-2 test result to surgery was found to be associated with a decreasing incidence of the composite outcome (aOR, 0.98 [per 10 days]; 95% CI, 0.97-1.00; *P* = .009) for the symptomatic subgroup (eTable 6 in Supplement 1). Increasing time interval from a positive SARS-CoV-2 test result to surgery showed a tendency toward decreasing risk of the composite outcome for the asymptomatic subgroup (aOR, 0.98 [per 10 days]; 95% CI, 0.96-1.00; *P* = .06 (eTable 7 in Supplement 1), although this finding did not reach the threshold for statistical significance.

No substantial change was observed in the estimates of association between the subgroup receiving at least 1 dose of a vaccine (1552 patients; aOR, 0.98 [per 10 days]; 95% CI, 0.97-1.00; *P* = .04) (eTable 8 in Supplement 1) and the unvaccinated subgroup (2445 cases; aOR, 0.98 [per 10 days]; 95% CI, 0.97-1.00; *P* = .02) (eTable 9 in Supplement 1).

## Discussion

The COVID-19 pandemic has had an immense effect on our health care system. The complications of COVID-19 have been well described and include DVT, PE, CVA, myocardial injury, AKI, and death.^16,17,18^ Patients with previous COVID-19 infection have increased odds of these complications after undergoing surgery.^19^ The importance of this cannot be underscored enough as these complications have independently been shown to be associated with increased mortality and costs of care.^20,21,22,23^ The present study demonstrates that an increasing time interval between COVID-19 infection and surgery was associated with a decreasing risk of postoperative cardiovascular complications, including DVT, PE, CVA, myocardial injury, AKI, or death within 30 days of surgery. This association remained in multiple sensitivity analyses accounting for competing risk of death, pulmonary complications, severity of symptoms, and vaccination status.

Our results are congruent with those of earlier studies that have shown a postoperative increase in the incidence of morbidity among patients with COVID-19. This study expands on earlier work by using a large, single-center database to examine both recent infections (within 7-10 days) and more remote occurrences in a disease that may have serious long-term sequalae.^24^ Use of a composite outcome was an important addition to the literature to allow physicians to contextualize overall perioperative risk, which is a necessary step in perioperative planning.

This study aimed to illustrate the temporal association between COVID-19 infection and surgical timing with postoperative complications to better inform shared decision-making around delaying or proceeding with surgery after COVID-19 infection. Recent publications have cited general guidelines for specific postoperative complications such as major adverse cardiac events, DVT, and bleeding.^25,26^ Most recommend delaying surgery for at least 7 weeks after COVID-19 infection. This recommendation, however, is based on very limited evidence and it remains unclear how patient vaccination status or specific COVID-19 strains may be associated with these risks.^27^ Our findings provide a more granular exploration of the time-dependent association between COVID-19 infection and outcomes after surgery, estimating a 1% reduction in the risk of our composite outcome for every 10 days after diagnosis.

The highly variable presentation and long-term effects of COVID-19 infections have also led investigators to question the association between asymptomatic vs symptomatic infections and outcomes after surgery. Although one smaller observational study found an increased risk of mortality and pulmonary complications in asymptomatic surgical patients,^28^ results were not replicated in a larger cohort of asymptomatic patients presenting for elective surgery.^27^ Our study builds on this work by examining a large population and evaluating a wide array of significant postoperative complications. We did not find a statistically significant temporal association between the timing of surgery and postoperative outcomes among patients who were asymptomatic at the time of preoperative testing (aOR, 0.98 [per 10 days]; 95% CI, 0.96-1.00; *P* = .06), although our results did show a trend toward reducing rates of complications with delaying time to surgery. Evidence remains inconclusive and further work is needed to understand the potential role of severity of illness in surgical outcomes after COVID-19 infection and the utility of performing preoperative COVID-19 testing for all surgical patients.^29^

### Strengths and Limitations

This study has some strengths, such as the inclusion of well-defined and rigorous outcomes to quantify multiple postoperative morbidities. The use of a composite outcome is a unique method to measure risk associated with COVID-19, which is critical when deciding perioperative recommendations. Inclusion of any patient with a positive test result, regardless of timing, makes these results more applicable to the late stage of the pandemic, when most COVID-19 infections have occurred outside of the 7- to 10-day infection window used in most initial perioperative studies.^30,31^ The requirement for a positive PCR test result allowed for a clear diagnosis as well as time frame; however, patients without a positive PCR test result who did have COVID-19 could have been missed. In addition, the use of a large database and robust informatics infrastructure with rigorous sensitivity analyses allowed for more continuous assessment of risk, rather than arbitrary cutoffs.

This study also had important limitations. First, because this is a retrospective study, we cannot assume causation, only association. In addition, the virus, care, and recommendations during this pandemic have been dynamic. As more treatments are available and variants in the virus become evident, an infection in 2020 is most likely different than an infection in 2022. Although we were not able to stratify patients by specific variants, infection dates, or vaccination schedules, we have chosen to include as broad of a patient population as possible with no exclusion criteria for this initial assessment. Further studies and assessments will be necessary for this ever-changing disease. Capturing postoperative DVT and PE through *ICD-10* codes had the potential for inaccuracies, based on the unreliable nature of clinicians recording data in the medical record and the large variety of *ICD-10* codes for acute embolus. This limitation was mitigated by requiring both *ICD-10* codes and imaging study for DVT and PE, as well as extensive manual review and validation. Our definition of CVA—patients with a neurology consultation and stroke imaging performed—did not include confirmed stroke. This decision was made given the significant false-positive rate from manual review of the patients with an *ICD-10* code for preexisting stroke and evidence that this does not appear to be a valid means for detecting stroke in a retrospective observational study at our institution. Additional work is needed to improve the retrospective capture of CVA in observational data sets, given the importance of this outcome. The use of troponinemia as a surrogate for myocardial injury is imperfect but has been used in previous studies for myocardial injury after noncardiac surgery and myocardial injury with COVID-19 infection.^32,33^ Most patients did not have preoperative serum troponin levels measured for comparison; however, elevated serum troponin preoperatively would likely be seen only among patients with reduced kidney function.^34^ The original myocardial injury after noncardiac surgery study excluded patients with elevated serum troponin secondary to pulmonary embolism, sepsis, and cardioversion.^7^ The decision was made a priori to not exclude these patients for the analysis because they accounted for a negligible number of patients in the original study. Major adverse cardiac events were not used as there is no accepted universal definition.^35^

There were also several limitations resulting from the rapidly changing nature of care during this recent pandemic. We lacked the data to stratify patients based on partial vs full vaccination course or type of vaccine received. We were not able to investigate different strains of the virus as our hospital does not perform variant testing and we were not sufficiently powered to study temporal trends in our data set. The association of various COVID-19 strains with outcomes is an important area for future research at sites that perform routine variant testing. We may underreport the outcomes of interest if patients presented to non-VUMC hospitals or clinics with these complications. We were unable to ascertain the severity of COVID-19 infection from our data, and future studies may add value by addressing long-term implications of more severe infections. We were, however, able to stratify between asymptomatic and symptomatic infections. We were also unable to perform a case-by-case analysis of the urgency of each surgical procedure, although we did include ASA emergency status and surgical case acuity as covariates to try to account for urgency. Finally, patients in our study population often have preoperative comorbidities that place them at higher risk for individual outcomes that were used in the composite postoperative outcomes.

## Conclusions

The results of this cohort study suggest that there was a time-dependent association between time from COVID-19 diagnosis to surgical intervention and a composite outcome of DVT, PE, CVA, myocardial injury, AKI, or death. Understanding the potential benefits associated with delaying surgery provides a key step in clinicians’ ability to optimize surgical timing for the increasing population of patients who have been infected with COVID-19. Additional research is needed to determine the effect of the dynamic management of this disease and of newer COVID-19 variants on the association we have detected.

## Notes

Supplement 1. **eTable 1.** Multivariable Logistic Regression Model for the Analysis With Postoperative Myocardial Injury as Outcome **eTable 2.** Multivariable Logistic Regression Model for the Analysis With Postoperative Acute Kidney Injury as Outcome **eTable 3.** Multivariable Logistic Regression Model for the Analysis With 30-Day Mortality as Outcome **eTable 4.** Multivariable Logistic Regression Model for the Analysis on Primary Outcome After Excluding Death Cases **eTable 5.** Multivariable Logistic Regression Model for the Analysis on Primary Outcome and Postoperative Pulmonary Complications **eTable 6.** Multivariable Logistic Regression Model for the Analysis on Primary Outcome for Symptomatic Subgroup **eTable 7.** Multivariable Logistic Regression Model for the Analysis on Primary Outcome for Asymptomatic Subgroup (Forcing Exposure Variable to be Included) **eTable 8.** Multivariable Logistic Regression Model for the Analysis on Primary Outcome for Vaccinated Subgroup **eTable 9.** Multivariable Logistic Regression Model for the Analysis on Primary Outcome for Unvaccinated Subgroup **eAppendix.** Demographic Characteristics of the Study Sample by Time From Confirmed COVID-19 Diagnosis to Surgery

Supplement 2.
Data Sharing Statement
