# Supplementary material for: Association of Time to Surgery After COVID-19 Infection With Risk of Postoperative Cardiovascular Morbidity
Source: JAMA Netw Open. 2022 Dec 14;5(12):e2246922. doi: 10.1001/jamanetworkopen.2022.46922 (PMC9856239; doi:10.1001/jamanetworkopen.2022.46922)
Supplement: Supplement 1. — eTable 1. Multivariable Logistic Regression Model for the Analysis With Postoperative Myocardial Injury as Outcome eTable 2. Multivariable Logistic Regression Model for the Analysis With Postoperative Acute Kidney Injury as Outcome eTable 3. Multivariable Logistic Regression Model for the Analysis With 30-Day Mortality as Outcome eTable 4. Multivariable Logistic Regression Model for the Analysis on Primary Outcome After Excluding Death Cases eTable 5. Multivariable Logistic Regression Model for the Analysis on Primary Outcome and Postoperative Pulmonary Complications eTable 6. Multivariable Logistic Regression Model for the Analysis on Primary Outcome for Symptomatic Subgroup eTable 7. Multivariable Logistic Regression Model for the Analysis on Primary Outcome for Asymptomatic Subgroup (Forcing Exposure Variable to be Included) eTable 8. Multivariable Logistic Regression Model for the Analysis on Primary Outcome for Vaccinated Subgroup eTable 9. Multivariable Logistic Regression Model for the Analysis on Primary Outcome for Unvaccinated Subgroup eAppendix. Demographic Characteristics of the Study Sample by Time From Confirmed COVID-19 Diagnosis to Surgery [file jamanetwopen-e2246922-s001.pdf]

Supplementary Online Content

Bryant JM, Boncyk CS, Rengel KF, et al. Association of time to surgery after COVID-19 infection with risk of postoperative cardiovascular morbidity. *JAMA Netw Open.* 2022;5(12):e2246922. doi:10.1001/jamanetworkopen.2022.46922

eTable 1. Multivariable Logistic Regression Model for the Analysis With Postoperative Myocardial Injury as Outcome
eTable 2. Multivariable Logistic Regression Model for the Analysis With Postoperative Acute Kidney Injury as Outcome
eTable 3. Multivariable Logistic Regression Model for the Analysis With 30-Day Mortality as Outcome
eTable 4. Multivariable Logistic Regression Model for the Analysis on Primary Outcome After Excluding Death Cases
eTable 5. Multivariable Logistic Regression Model for the Analysis on Primary Outcome and Postoperative Pulmonary Complications
eTable 6. Multivariable Logistic Regression Model for the Analysis on Primary Outcome for Symptomatic Subgroup
eTable 7. Multivariable Logistic Regression Model for the Analysis on Primary Outcome for Asymptomatic Subgroup (Forcing Exposure Variable to be Included)
eTable 8. Multivariable Logistic Regression Model for the Analysis on Primary Outcome for Vaccinated Subgroup
eTable 9. Multivariable Logistic Regression Model for the Analysis on Primary Outcome for Unvaccinated Subgroup
eAppendix. Demographic Characteristics of the Study Sample by Time From Confirmed COVID-19 Diagnosis to Surgery

This supplementary material has been provided by the authors to give readers additional information about their work.

© 2022 Bryant JM et al. *JAMA Network Open.*

eTable 1. Multivariable Logistic Regression Model for Analysis With Postoperative Myocardial Injury as Outcome

Outcomes	Variables	Adjusted Odds Ratio with 95% CI	*P*-value
Myocardial Injury	Time interval from COVID positive diagnosis to surgery (per 10 days increase)	0.97 (0.95 to 0.99)	0.004
	Age (per 10 years older)	1.30 (1.12 to 1.50)	<.001
	ASA classification	2.47 (1.70 to 3.59)	<.001
	Case acuity (emergency *versus* elective)	1.89 (1.07 to 3.33)	0.03
	Surgical duration (per 1 hour increase)	1.08 (1.01 to 1.16)	0.03
	Elixhauser comorbidity Congestive Heart Failure	2.35 (1.44 to 3.81)	<.001
	Elixhauser comorbidity Cardiac Arrhythmias	5.29 (3.06 to 9.16)	<.001
	Elixhauser comorbidity Pulmonary Circulation Disorders	3.40 (1.80 to 6.41)	<.001
	Elixhauser comorbidity Hypertension, Complicated	2.07 (1.25 to 3.43)	0.005
	Elixhauser comorbidity Liver Disease	2.73 (1.52 to 4.89)	<.001
	Elixhauser comorbidity Metastatic Cancer	3.51 (1.62 to 7.61)	0.002
	Elixhauser comorbidity Blood Loss Anemia	4.15 (1.65 to 10.44)	0.003

© 2022 Bryant JM et al. *JAMA Network Open.*

eTable 2. Multivariable Logistic Regression Model for the Analysis With Postoperative Acute Kidney Injury as Outcome

Outcome	Variables	Adjusted Odds Ratio with 95% CI	*P*-value
Acute Kidney Injury	Time interval from COVID positive diagnosis to surgery (per 10 days increase)	0.99 (0.98 to 1.00)	0.04
	Age (per 10 years older)	1.12 (1.03 to 1.21)	0.009
	Male gender	1.57 (1.20 to 2.05)	0.001
	Race Black / African American (vs race White)	2.17 (1.59 to 2.97)	<.001
	ASA classification	2.11 (1.70 to 2.63)	<.001
	Intracranial neurosurgical procedure	0.22 (0.07 to 0.72)	0.01
	Urologic procedure	2.14 (1.46 to 3.13)	<.001
	Elixhauser comorbidity Cardiac Arrhythmias	1.43 (1.06 to 1.93)	0.02
	Elixhauser comorbidity Renal Failure	3.19 (2.39 to 4.27)	<.001
	Elixhauser comorbidity Liver Disease	1.72 (1.14 to 2.59)	0.01
	Elixhauser comorbidity Solid Tumor	1.89 (1.30 to 2.75)	0.001
	Elixhauser comorbidity Coagulopathy	1.74 (1.15 to 2.75)	0.009
	Elixhauser comorbidity Fluid & Electrolyte Disorders	3.09 (2.26 to 4.24)	<.001

© 2022 Bryant JM et al. *JAMA Network Open.*

eTable 3. Multivariable Logistic Regression Model for the Analysis With 30-Day Mortality as Outcome

**Outcome**	**Variables**	**Adjusted Odds Ratio with 95% CI**	***P*-value**
30-day Mortality	Time interval from COVID positive diagnosis to surgery (per 10 days increase)	0.96 (0.93 to 0.99)	0.007
	Age (per 10 years older)	1.36 (1.15 to 1.61)	<.001
	Male gender	2.29 (1.24 to 4.25)	0.008
	ASA classification	2.55 (1.62 to 4.02)	<.001
	Case acuity (emergency *versus* elective)	2.17 (1.15 to 4.08)	0.02
	Elixhauser comorbidity Cardiac Arrhythmias	2.18 (1.13 to 4.18)	0.02
	Elixhauser comorbidity Metastatic Cancer	5.35 (2.40 to 11.91)	<.001
	Elixhauser comorbidity Coagulopathy	3.63 (2.05 to 6.42)	<.001
	Elixhauser comorbidity Fluid & Electrolyte Disorders	4.42 (2.29 to 8.52)	<.001

© 2022 Bryant JM et al. *JAMA Network Open.*

eTable 4. Multivariable Logistic Regression Model for the Analysis on Primary Outcome after Excluding Death Cases

Outcome	Variables	Adjusted Odds Ratio with 95% CI	*P*-value
Primary composite outcome of postoperative DVT, PE, CVA, myocardial injury, and AKI within 30 days following surgery	Time interval from COVID positive diagnosis to surgery (per 10 days increase)	0.99 (0.98 to 1.00)	0.007
	Age (per 10 years older)	1.11 (1.03 to 1.20)	0.009
	Male gender	1.35 (1.05 to 1.75)	0.02
	Race black or African American (*versus* race white)	2.02 (1.50 to 2.73)	<.001
	ASA classification	2.41 (1.94 to 2.99)	<.001
	Urologic procedure	2.14 (1.48 to 3.11)	<.001
	Elixhauser comorbidity Cardiac Arrhythmias	1.96 (1.47 to 2.60)	<.001
	Elixhauser comorbidity Paralysis	2.10 (1.05 to 4.18)	0.04
	Elixhauser comorbidity Neurodegenerative Disorders	1.93 (1.13 to 3.32)	0.02
	Elixhauser comorbidity Renal Failure	2.92 (2.19 to 3.91)	<.001
	Elixhauser comorbidity Solid Tumor	2.00 (1.38 to 2.92)	<.001
	Elixhauser comorbidity Coagulopathy	2.12 (1.38 to 3.25)	<.001
	Elixhauser comorbidity Weight Loss	1.69 (1.12 to 2.54)	0.01
	Elixhauser comorbidity Fluid & Electrolyte Disorders	2.44 (1.79 to 3.33)	<.001
	Elixhauser comorbidity Psychoses	2.61 (1.15 to 5.92)	0.02

© 2022 Bryant JM et al. *JAMA Network Open.*

eTable 5. Multivariable Logistic Regression Model for the Analysis on Primary Outcome and Postoperative Pulmonary Complications

Outcome	Variables	Adjusted Odds Ratio with 95% CI	*P*-value
Primary composite outcome of postoperative DVT, PE, CVA, myocardial injury, AKI, death, and PPCs within 30 days following surgery	Time interval from COVID positive diagnosis to surgery (per 10 days increase)	0.99 (0.98 to 1.00)	0.002
	Male gender	1.38 (1.10 to 1.74)	0.005
	Race Black / African American (vs race White)	1.62 (1.23 to 2.14)	<.001
	ASA classification	3.01 (2.48 to 3.65)	<.001
	Urologic procedure	1.94 (1.35 to 2.78)	<.001
	Elixhauser comorbidity Cardiac Arrhythmias	2.16 (1.68 to 2.78)	<.001
	Elixhauser comorbidity Pulmonary Circulation Disorders	2.84 (1.55 to 5.22)	<.001
	Elixhauser comorbidity Paralysis	2.63 (1.38 to 5.02)	0.003
	Elixhauser comorbidity Renal Failure	2.12 (1.61 to 2.80)	<.001
	Elixhauser comorbidity Lymphoma	3.90 (1.44 to 10.53)	0.007
	Elixhauser comorbidity Solid Tumor	2.12 (1.50 to 2.98)	<.001
	Elixhauser comorbidity Coagulopathy	1.97 (1.31 to 2.98)	0.001
	Elixhauser comorbidity Weight Loss	1.95 (1.32 to 2.88)	<.001
	Elixhauser comorbidity Fluid & Electrolyte Disorders	3.38 (2.57 to 4.45)	<.001

© 2022 Bryant JM et al. *JAMA Network Open.*

eTable 6. Multivariable Logistic Regression Model for the Analysis on Primary Outcome for Symptomatic Subgroup

Outcome	Variables	Adjusted Odds Ratio with 95% CI	*P*-value
Primary composite outcome of postoperative DVT, PE, CVA, myocardial injury, AKI, and death within 30 days following surgery	Time interval from COVID positive diagnosis to surgery (per 10 days increase)	0.98 (0.97 to 1.00)	0.009
	Age (per 10 years older)	1.20 (1.07 to 1.33)	0.001
	Race Black / African American (vs race white)	1.83 (1.22 to 2.75)	0.003
	Race Others (vs race White)	2.45 (1.37 to 4.39)	0.003
	ASA classification	2.60 (1.93 to 3.51)	<.001
	Urologic procedure	2.90 (1.76 to 4.77)	<.001
	Elixhauser comorbidity Cardiac Arrhythmias	2.10 (1.43 to 3.08)	<.001
	Elixhauser comorbidity Renal Failure	2.49 (1.69 to 3.66)	<.001
	Elixhauser comorbidity Metastatic Cancer	5.49 (2.77 to 10.89)	<.001
	Elixhauser comorbidity Coagulopathy	2.51 (1.43 to 4.41)	0.001
	Elixhauser comorbidity Weight Loss	2.03 (1.17 to 3.50)	0.01
	Elixhauser comorbidity Fluid & Electrolyte Disorders	3.22 (2.13 to 4.87)	<.001
	Elixhauser comorbidity Psychoses	9.32 (1.35 to 64.20)	0.02

© 2022 Bryant JM et al. *JAMA Network Open.*

eTable 7. Multivariable Logistic Regression Model for the Analysis on Primary Outcome for Asymptomatic Subgroup (Forcing Exposure Variable to be Included)

Outcome	Variables	Adjusted Odds Ratio with 95% CI	*P*-value
Primary composite outcome of postoperative DVT, PE, CVA, myocardial injury, AKI, and death within 30 days following surgery	Time interval from COVID positive diagnosis to surgery (per 10 days increase)	0.98 (0.96 to 1.00)	0.06
	Male gender	2.09 (1.44 to 3.04)	<.001
	Race Black / African American (vs race White)	2.20 (1.43 to 3.39)	<.001
	ASA classification	2.64 (1.97 to 3.53)	<.001
	Elixhauser comorbidity Cardiac Arrhythmias	2.08 (1.42 to 3.06)	<.001
	Elixhauser comorbidity Diabetes, Complicated	1.53 (0.96 to 2.44)	0.07
	Elixhauser comorbidity Renal Failure	2.93 (1.85 to 4.65)	<.001
	Elixhauser comorbidity Coagulopathy	2.37 (1.31 to 4.28)	0.004
	Elixhauser comorbidity Weight Loss	2.36 (1.35 to 4.12)	0.003
	Elixhauser comorbidity Fluid & Electrolyte Disorders	2.11 (1.38 to 3.22)	<.001

© 2022 Bryant JM et al. *JAMA Network Open.*

**eTable 8.** Multivariable Logistic Regression Model for the Analysis on Primary Outcome for Vaccinated Subgroup			
**Outcome**	**Variables**	**Adjusted Odds Ratio with 95% CI**	***P*-value**
Primary composite outcome of postoperative DVT, PE, CVA, myocardial injury, AKI, and death within 30 days following surgery	Time interval from COVID positive diagnosis to surgery (per 10 days increase)	0.98 (0.97 to 1.00)	0.04
	Male gender	1.52 (1.01 to 2.31)	0.05
	Race Black / African American (vs race White)	1.94 (1.17 to 3.22)	0.01
	ASA classification	3.07 (2.12 to 4.46)	<.001
	ASA emergency status	2.66 (1.19 to 5.92)	0.02
	Urologic procedure	2.38 (1.33 to 4.27)	0.004
	Elixhauser comorbidity Congestive Heart Failure	2.14 (1.27 to 3.63)	0.005
	Elixhauser comorbidity Renal Failure	2.76 (1.73 to 4.40)	<.001
	Elixhauser comorbidity Lymphoma	24.09 (4.98 to 116.63)	<.001
	Elixhauser comorbidity Solid Tumor	2.78 (1.57 to 4.92)	<.001
	Elixhauser comorbidity Coagulopathy	2.55 (1.11 to 5.88)	0.03
	Elixhauser comorbidity Fluid & Electrolyte Disorders	2.74 (1.64 to 4.57)	<.001

© 2022 Bryant JM et al. *JAMA Network Open.*

eTable 9. Multivariable Logistic Regression Model for the Analysis on Primary Outcome for Unvaccinated Subgroup

Outcome	Variables	Adjusted Odds Ratio with 95% CI	*P*-value
Primary composite outcome of postoperative DVT, PE, CVA, myocardial injury, AKI, and death within 30 days following surgery	Time interval from COVID positive diagnosis to surgery (per 10 days increase)	0.98 (0.97 to 1.00)	0.02
	Age (per 10 years older)	1.14 (1.04 to 1.25)	0.004
	Male gender	1.53 (1.12 to 2.08)	0.007
	Race Black / African American (vs race White)	1.90 (1.32 to 2.74)	<.001
	ASA classification	2.22 (1.73 to 2.85)	<.001
	Urologic procedure	1.76 (1.08 to 2.86)	0.02
	Elixhauser comorbidity Cardiac Arrhythmias	2.33 (1.67 to 3.25)	<.001
	Elixhauser comorbidity Paralysis	2.32 (1.09 to 4.97)	0.03
	Elixhauser comorbidity Neurodegenerative Disorders	2.29 (1.22 to 4.29)	0.01
	Elixhauser comorbidity Renal Failure	2.63 (1.83 to 3.78)	<.001
	Elixhauser comorbidity Solid Tumor	1.90 (1.21 to 3.01)	0.006
	Elixhauser comorbidity Coagulopathy	2.62 (1.65 to 4.16)	<.001
	Elixhauser comorbidity Weight Loss	1.83 (1.16 to 2.89)	0.01
	Elixhauser comorbidity Fluid & Electrolyte Disorders	2.66 (1.87 to 3.80)	<.001

© 2022 Bryant JM et al. *JAMA Network Open.*

**eAppendix.** Demographic Characteristics of the Study Sample by Time From Confirmed COVID-19 Diagnosis to Surgery				
	**Time from Confirmed COVID-19 Diagnosis to Surgery**			
**Variables**	**Less than 30 days (N = 990)**	**Between 30 and 98 days (N = 1,006)**	**Between 98 and 225 days (N = 997)**	**Greater than 225 days (N = 1,004)**
**Age** in years, median (IQR)	47.0 (29.8-64.1)	51.6 (36.1-62.9)	52.1 (38.1-65.5)	52.3 (37.7-65.1)
**Body Mass Index** in kg/m^2, median (IQR)	29.0 (25.0-34.0)	29.0 (25.0-35.0)	29.0 (25.0-34.0)	29.0 (25.0-35.0)
**Sex** (%)				
Female	521 (52.6%)	573 (57.0%)	539 (54.1%)	590 (58.8%)
**Race** (%)				
African American/Black	156 (15.6%)	162 (16.1%)	183 (18.4%)	166 (16.7%)
White	721 (72.8%)	757 (75.3%)	744 (74.6%)	768 (76.5%)
Other[1]	113 (11.4%)	87 (8.7%)	70 (7.0%)	70 (7.0%)
**ASA Physical Status Classification** (%)				
1	40 (4.0%)	45 (4.5%)	51 (5.1%)	57 (5.7%)
2	289 (29.2%)	321 (31.9%)	336 (33.7%)	379 (37.8%)
3	495 (50.0%)	520 (51.7%)	506 (50.8%)	473 (47.1%)
4	152 (15.4%)	119 (11.8%)	103 (10.3%)	95 (9.5%)
5	14 (1.4%)	1 (0.1%)	-	-
6	-	-	1 (0.1%)	-

[1] The "Other" race category was defined as all races other than African American/Black and White.

© 2022 Bryant JM et al. *JAMA Network Open.*

**ASA Emergency** (%)				
Yes	210 (21.2%)	27 (2.7%)	18 (1.8%)	22 (2.2%)
**Case Acuity** (%)				
Elective	784 (79.2%)	968 (96.2%)	972 (97.5%)	977 (97.3%)
Emergency	206 (20.8%)	38 (3.8%)	25 (2.5%)	27 (2.7%)
**Duration of Surgery** in hours, median (IQR)	2.5 (1.5-4.0)	1.3 (0.6-2.7)	1.2 (0.6-2.5)	1.2 (0.5-2.6)
**Incidence of DVT** (%)	37 (3.7%)	8 (0.8%)	9 (0.9%)	7 (0.7%)
**Incidence of PE** (%)	7 (0.7%)	4 (0.4%)	3 (0.3%)	2 (0.2%)
**Incidence of CVA** (%)	20 (2.0%)	5 (0.5%)	2 (0.2%)	2 (0.2%)
**Incidence of Myocardial Injury** (%)	55 (5.6%)	28 (2.8%)	20 (2.0%)	13 (1.3%)
**Incidence of AKI** (%)	122 (12.3%)	93 (9.2%)	74 (7.4%)	74 (7.4%)
**30-day Mortality** (%)	48 (4.9%)	15 (1.5%)	13 (1.3%)	3 (0.3%)
**Incidence of Primary Composite Outcome** (%)	183 (18.5%)	121 (12.0%)	95 (9.5%)	86 (8.6%)

© 2022 Bryant JM et al. *JAMA Network Open.*
