# Supplementary material for: Association of Time to Surgery After COVID-19 Infection With Risk of Postoperative Cardiovascular Morbidity
Source: JAMA Netw Open. 2022 Dec 14;5(12):e2246922. doi: 10.1001/jamanetworkopen.2022.46922 (PMC9856239; doi:10.1001/jamanetworkopen.2022.46922)
Supplement: Supplement 2. — Data Sharing Statement [file jamanetwopen-e2246922-s002.pdf]

Data Sharing Statement

Bryant. Association of Time to Surgery After COVID-19 Infection With Risk of Postoperative Cardiovascular Morbidity. *JAMA Netw Open*. Published December 14, 2022. doi:10.1001/jamanetworkopen.2022.46922

Data
Data available: No

Additional Information
Explanation for why data not available: We do not have IRB approval to share data
